# Advances in the genetic dissection of plant cell walls: tools and resources available in *Miscanthus*

**DOI:** 10.3389/fpls.2013.00217

**Published:** 2013-07-04

**Authors:** Gancho Slavov, Gordon Allison, Maurice Bosch

**Affiliations:** Institute of Biological, Environmental and Rural Sciences, Aberystwyth UniversityCeredigion, UK

**Keywords:** *Miscanthus*, cell wall, recalcitrance, chemotyping, QTL, GWAS, functional genomics, genetic engineering

## Abstract

Tropical C_4_ grasses from the genus *Miscanthus* are believed to have great potential as biomass crops. However, *Miscanthus* species are essentially undomesticated, and genetic, molecular and bioinformatics tools are in very early stages of development. Furthermore, similar to other crops targeted as lignocellulosic feedstocks, the efficient utilization of biomass is hampered by our limited knowledge of the structural organization of the plant cell wall and the underlying genetic components that control this organization. The Institute of Biological, Environmental and Rural Sciences (IBERS) has assembled an extensive collection of germplasm for several species of *Miscanthus*. In addition, an integrated, multidisciplinary research programme at IBERS aims to inform accelerated breeding for biomass productivity and composition, while also generating fundamental knowledge. Here we review recent advances with respect to the genetic characterization of the cell wall in *Miscanthus*. First, we present a summary of recent and on-going biochemical studies, including prospects and limitations for the development of powerful phenotyping approaches. Second, we review current knowledge about genetic variation for cell wall characteristics of *Miscanthus* and illustrate how phenotypic data, combined with high-density arrays of single-nucleotide polymorphisms, are being used in genome-wide association studies to generate testable hypotheses and guide biological discovery. Finally, we provide an overview of the current knowledge about the molecular biology of cell wall biosynthesis in *Miscanthus* and closely related grasses, discuss the key conceptual and technological bottlenecks, and outline the short-term prospects for progress in this field.

## Background

Despite the wide variability of quantitative estimates, studies aimed at assessing bioenergy potentials consistently suggest that lignocellulosic biomass is expected to become a major global source of renewable energy, thereby significantly reducing dependency on fossil fuels (Berndes et al., 2003; Sims et al., 2006; Carroll and Somerville, 2009; Bentsen and Felby, 2012). As a result of substantial government support and interest from the private sector (Sims et al., 2010), active research programmes worldwide are targeting a large number of phylogenetically and ecologically diverse plant species, and genomics approaches are playing an increasingly prominent role in informing crop development (Rubin, 2008; Feltus and Vandenbrink, 2012). However, making products derived from second-generation (i.e., lignocellulose-based) energy crops economically competitive will require major breakthroughs both in increasing dry biomass yields on low-value lands and in optimizing the efficiency of biomass conversion procedures (Sims et al., 2010; Feltus and Vandenbrink, 2012).

Tropical C_4_ grasses from the genus *Miscanthus* are among the most promising second-generation energy crops (Carroll and Somerville, 2009; Feltus and Vandenbrink, 2012). This is because of their potentially very high productivity and low requirements for agricultural inputs (Clifton-Brown et al., 2004, 2007). Several public and private breeding programmes in East Asia, Europe and North America are therefore aiming to create *Miscanthus* hybrids and varieties that (i) are high-yielding and well-adapted to a range of climatic and edaphic conditions, (ii) can be established at low costs (i.e., ideally by seed) and (iii) have optimal cell wall characteristics for conversion. Because progress with the fundamental understanding and modeling of the former two groups of traits has recently been summarized elsewhere (e.g., Hastings et al., 2009; Clifton-Brown et al., 2011; Jensen et al., 2011, 2013; Robson et al., 2011, 2012), this review will focus on current knowledge about cell wall biosynthesis in *Miscanthus* and the prospects of developing detailed molecular genetic and biochemical models that would help reduce recalcitrance to biomass conversion.

## Cell wall biochemistry and phenotyping

### Grass cell walls

Cell walls are strong flexible composites of polymers that maintain the structural integrity of the cell. Most plant biomass, consisting primarily of cellulose, hemicellulose and lignin, contains on average approximately 30–50%, 10–40%, and 5–30% by weight of these cell wall polymers, respectively (McKendry, 2002). The primary cell wall is laid down outside of the cell membrane as a layered structure, and secondary cell wall thickening occurs between the primary wall and the cell membrane. The primary cell wall is thin and flexible, allowing new growth and cell extension to take place. Primary cell walls of grasses contain higher proportions of cellulose, negligible amounts of pectin (1–2%), and substantial amounts of cell wall-bound hydroxycinnamic acids (up to 3% of *p*- coumaric acid and up to 4% of ferulic acid) compared to dicot species (Grabber et al., 1995; Waldron et al., 1996; Vogel, 2008; Allison et al., 2009b). Cellulose, in the form of microfibrils, is embedded in a hydrated matrix of hemicelluloses. Cellulose microfibrils can be exceedingly long and are formed from many semi-crystalline chains, each comprising 500–14,000 D-glucose monomers joined linearly by β_1−4_ linkages (Delmer and Amor, 1995).

Hemicelluloses are a heterogeneous group of polysaccharides that have β-(1→4)-linked backbones of glucose, mannose, or xylose. Their structure and abundance can vary widely between different species and cell types, but their predominant role is to strengthen the cell wall by interaction with cellulose and lignin (Scheller and Ulvskov, 2010). The predominant hemicellulose in secondary walls of grasses is glucuronoarabinoxylan (GAX), which may comprise 40–50% of cell wall polysaccharides by weight in the grass secondary wall and ~20% of total grass biomass (Carpita and Gibeaut, 1993; Scheller and Ulvskov, 2010; Kulkarni et al., 2012). In GAX, the xylan backbone is substituted with arabinose and to a much lesser degree, 4-O-Methyl gluconate and gluconic acid, which may be esterified to lignin (Sun et al., 2005). Another important aspect of these hemicelluloses is that ferulic acid is attached to GAX by ester linkages between its carboxyl group and the C5-hydroxyl of arabinofuranosyl residues. Some ferulic acid moieties exist as a variety of dimers that serve to cross-link GAX chains through inter- and intra-molecular bonds (Grabber et al., 1998a,b; Akin, 2007). Ferulates also cross-link through ether bonds to lignin, where they act as nucleation sites for lignin polymerization during lignification (Ralph et al., 1995; Hatfield et al., 1999b). These features result in a highly cross-linked and recalcitrant matrix involving carbohydrates and lignin.

Secondary walls, which are laid down during the differentiation of xylem, phloem and transfer cells after elongation, are generally thicker than primary walls, and most importantly, lignin replaces much of the water that is present in primary cell walls, making secondary cell walls impenetrable to solutes and enzymes (Pauly and Keegstra, 2008). Lignin is a complex aromatic heteropolymer and is often highest in concentration in the vascular tissues. It is covalently bound to hemicellulose and gives the strength and rigidity that terrestrial plants need to grow upright. It also provides hydrophobicity to the vascular system, a prerequisite for the effective transport of water and solutes (Vanholme et al., 2008). Lignin consists primarily of three hydroxycinnamyl alcohol monolignol monomers (hydroxyphenyl/guaiacyl/syringyl; H/G/S) that differ in their degree of methoxylation (Boudet, 1998; Boerjan et al., 2003) and are polymerized into a highly complex and somewhat random structure by ether and carbon-carbon bonds. Current opinion holds that lignin biosynthesis occurs in the extracellular milieu, where the monolignols are oxidized by peroxide or laccase enzymes and coupled in a combinatorial fashion (Morreel et al., 2004; Barsberg et al., 2006; Méchin et al., 2007).

### The effect of cell wall composition on conversion efficiency

The majority of energy stored in biomass is contained within the dense polymers of the cell wall, which is also the major component of dried biomass by weight. Wood biomass typically has a water content of 10–20% after seasoning or drying and consists almost entirely of cell wall. Harvested biomass from energy grass species, including *Miscanthus*, generally contains 70–90% cell wall (Allison et al., 2010; Hodgson et al., 2010a,b), and even forage grass biomass is comprised of approximately 50% cell wall (Wilman and Rezvani, 1998).

The concentration and composition of the cell wall affects not only the digestibility of biomass, when fed as forage to cattle and sheep (Jung and Fahey, 1983; Jung and Buxtono, 1994; Hatfield et al., 1999c), but also recalcitrance to enzymatic deconstruction (saccharification) (Akin, 2007, 2008; Ding et al., 2012) and fermentation to bioethanol and other products (Affeltranger and Filer, 1986; Klinke et al., 2004; Yee et al., 2012). Frequently, utilization of the fermentable sugars stored in the carbohydrate polymers of the cell wall is limited by the presence of lignin, and the reduction of lignin content will most likely be a central strategy for biomass improvement in many energy grass species (Boudet et al., 2003; Sticklen, 2006; Chang, 2007; Chen and Dixon, 2007; Li et al., 2008). In contrast, increasing lignin content will improve calorific value and energy density for thermochemical conversion (Fahmi et al., 2008; Allison et al., 2009a, 2010; Hodgson et al., 2010a,b). The improvement of biomass crops is therefore complex and requires thorough understanding of (i) cell wall composition and architecture, (ii) how changes in these parameters are likely to affect plant physiology, development and disease resistance, and (iii) how the biomass is to be utilized. Lignin is the most likely target for manipulation in the short to medium term as several studies in energy crops and related species have shown that lignin concentration and composition can be altered by mutation (Vignols et al., 1995; Halpin et al., 1998), breeding (Clifton-Brown et al., 2008), and transgenic intervention (Hu et al., 1999; Anterola and Lewis, 2002; Huntley et al., 2003; Vanholme et al., 2008; Grabber et al., 2010; Fu et al., 2011b; Yee et al., 2012).

Another potential target for compositional improvement of biomass feedstocks is the reduction of cellulose crystallinity. The exclusion of water and the steric hindrance imposed by the tightly packed glucan chains in crystalline cellulose limit access of hydrolytic enzymes to substrate and reduce saccharification efficiency (Jeoh et al., 2007; Hall et al., 2010). Whilst several pretreatment methods have been developed to disrupt cellulose crystallinity (Hendriks and Zeeman, 2009; Kumar et al., 2009; Chundawat et al., 2011), this parameter seems resistant to many pretreatment strategies (Puri, 1984), and a better strategy might be to decrease cellulose crystallinity in *Miscanthus* using genetic manipulation as has been demonstrated in *Arabidopsis* (Harris et al., 2009).

### Methods that measure cell wall composition

The measurement of cell wall composition is far from being a trivial matter due to its complexity, and most cell wall chemists employ an eclectic mix of modern and very traditional methods. In most cases, measurements are not wholly quantitative and often data is very dependent on the methods employed. However, the genetic dissection of cell wall composition will rely to a very large degree on the chemical analysis of samples, and an understanding of the strengths and weaknesses of the various approaches is going to be essential. Therefore, we next examine several of the main methods being used currently.

#### Gravimetric methods

Gravimetric, or direct methods, are based on the sequential extraction of plant material with acid and alkali solutions. The cell wall components can be classified by their stability to increasingly harsh chemical treatments. For example, hemicellulose is hydrolyzed by treatment with mild acid and alkali, whilst cellulose only succumbs to hydrolysis when treated with highly concentrated sulphuric acid. Lignin, in contrast, is mostly unaffected by hydrolysis under these conditions. The amount of each fiber fraction in the biomass sample is calculated from the decrease in sample weight following each treatment (Van Soest, 1967; Theander and Westerlund, 1986). Gravimetric methods are often complex, time consuming and costly (Theander and Westerlund, 1993; Giger-Reverdin, 1995). In addition, these methods are semi-quantitative as even mild acid or alkali treatments cause the partial degradation of non-target fractions.

The Weende method was the first standardized procedure and was developed at the Research Station of Weende in Germany in the 1860 s. Samples were boiled for 30 min in dilute sulphuric acid, followed by a second boiling step in dilute NaOH, resulting in the recovery of crude fiber comprised of cellulose, lignin, and the waxy epidermal polymers cutin and suberin. Soon it was recognized that this method had limitations as some lignin was dissolved by the extraction process, and the proportion of lignin lost varied considerably with the material being analysed.

Today the methods of Van Soest have largely replaced the Weende method; the first of these, the neutral detergent method (Van Soest, 1967) is used to isolate a total cell wall fraction (neutral detergent fiber, NDF) by boiling extraction with a detergent (sodium lauryl sulphate) at neutral pH. Hemicellulose is not hydrolyzed under these conditions, and with various modifications the method has been used extensively to estimate total cell wall in forages that contain little or no pectin. The method is less appropriate for measuring total cell wall in samples that contain pectin, which is effectively removed by the detergent. The acid detergent method of Van Soest (1963) has been used extensively to measure lignocellulose, which is extracted in dilute acid containing cetyltrimethylammonium bromide (CTAB). The resulting acid detergent fiber (ADF) is almost entirely composed of lignin and cellulose, and the method is relatively rapid and highly robust. A more advanced approach for the gravimetric analysis of the cell wall carbohydrate has been proposed by Theander and co-workers. Their unified Uppsala method (Theander and Westerlund, 1986, 1993) produces alcohol insoluble residues after extraction by sonication. Enzymes are then used to remove storage carbohydrate. This method does not result in the degradation of hemicellulose or pectin, but has the drawback that lignin may be overestimated because proteins are present in the final fiber fraction (Hatfield and Fukushima, 2005).

Gravimetric measurement of lignin is most commonly achieved by two methods. Analysis by the Klason method has long been the standard approach for use with wood. Samples are treated with 72% sulphuric acid to hydrolyze the structural carbohydrates, and the insoluble material left comprises lignin and ash (Kirk and Obst, 1988). Although some lignin may be lost in the process, protein, suberin and other components often condense and are counted as lignin. In samples with high protein content, this can lead to gross over-estimation. The acid detergent lignin (ADL) procedure (Van Soest, 1967) was developed as an alternative procedure for forages containing large amounts of protein. Consequently, the ADL method is used commonly in animal science (Jung et al., 1999). Even this method, however, is not immune to interference, and some plant metabolites that are resistant to acid and base hydrolysis, e.g., condensed tannins, interfere with lignin estimation (Makkar et al., 1995). The ADL method most likely underestimates lignin concentration in samples of grass cell wall, often by >50% compared to the Klason method, or alternative analytical methods such as measurement by the acetyl bromide method (Takahashi et al., 2004; Hatfield and Fukushima, 2005), and it is not known whether this is due to chemical or structural differences between wood and grass cell wall biomass. Measurement of the lost lignin can be made by UV spectrophotometry (TAPPI, 1999), but this is also prone to error because under strongly acidic conditions, pentose and hexose sugars may be converted to furfurals and hydroxymethyl furfurals, which like lignin absorb strongly in the UV (Hatfield and Fukushima, 2005). However, both methods correlate strongly with dry matter digestibility and may therefore be equally good predictors of feedstock utility for lignocellulosic processing (Jung, 1997).

#### Non-gravimetric methods

The ease by which hemicellulose can be isolated from the cell wall, or removed from cellulose, by water and alkali extraction is widely used to prepare samples for structural analysis by nuclear magnetic resonance and mid-infrared spectroscopy (Sun and Sun, 2002; Liu et al., 2006; Xu et al., 2006a,b; Samuel et al., 2009), but these approaches are generally not applicable for high-throughput analysis. More practical non-gravimetric approaches to measure the concentration of cell wall carbohydrates often rely on sequential treatments with specific hydrolases, after the removal of storage carbohydrates, such as starch (Selig et al., 2008). The high degree of selectivity exhibited by glycosidic enzymes allows a targeted degradation of the individual cell wall components, and subsequent chromatographic analysis provides information on the concentrations of individual carbohydrate fractions, their composition and the way in which the sugar monomers are linked. Until recently, the cost and time associated with these methods would have been prohibitive, but the availability of laboratory robots and affordable enzymes are making this kind of approach an increasingly feasible methodological choice (Foster et al., 2010b).

Non-gravimetric methods to measure lignin in wood are often based on the consumption of oxidants, typically chlorine or potassium permanganate. This approach is useful in the paper industry because it informs of lignin concentration and the bleaching requirement necessary to produce quality paper (Hatfield and Fukushima, 2005). More applicable to the analysis of biomass samples for crop improvement is lignin quantification using acetyl bromide. The method was first published by Johnson et al. (1961) and is based on the solubility of lignin at 50°C in acetyl bromide dissolved in concentrated acetic acid. Following solubilization, the polybromide anion that forms during the reaction is removed by reaction with hydroxylamine, and quantification of lignin concentration is made by relating absorbance at 280 nm to a standard curve obtained using lignin extracted by solubilization in acetyl bromide (Fukushima and Dehority, 2000) or acidic dioxane (Fukushima and Hatfield, 2001). Considerable care needs to be taken regarding experimental conditions as temperatures exceeding 50°C, or the addition of trichloroacetic acid, may lead to the degradation of hemicellulose and overestimation of lignin concentration (Hatfield et al., 1999a). The original method has been improved and used to measure lignin in a wide range of species, often from very small amounts of tissue (Fukushima and Dehority, 2000; Foster et al., 2010a). Several groups have recommended that for grass samples, the method can be improved still further by removing soluble and cell wall bound hydroxycinnamic acids in a preliminary incubation at alkaline pH (Brinkmann et al., 2002; Fukushima and Hatfield, 2004). However, Ralph et al. (1994, 1998) suggest that, as approximately half of total ferulates and nearly all of the p-coumaric acid are bound to lignin, these should be considered as part of the total macromolecule. The concentrations of lignin detected by the acetyl bromide approach are comparable with those obtained by Klason or permanganate determination (Hatfield and Fukushima, 2005), and values correlate strongly with dry matter digestibility (Fukushima and Dehority, 2000).

#### Pyrolytic methods

Analytical pyrolysis allows composition to be analysed in samples of biomass with minimal preparation, samples only requiring drying and milling. This approach has been used to characterize cell wall structure (Alves et al., 2006a,b; del Rio et al., 2007), explore variation in cell wall composition (Yokoi et al., 2001; Hodgson et al., 2010b) and investigate the influence of biomass feedstock composition and preparation during fast-pyrolysis (Bridgeman et al., 2007; Fahmi et al., 2007a, 2008; Yanik et al., 2007; Hodgson et al., 2010a). Analysis is achieved by placing the sample in a heating device in an oxygen-free atmosphere. The temperature is increased rapidly to the point where the sample decomposes by thermal fission into small molecules, and these are analysed by gas chromatography/mass spectrometry (GC/MS) (Galletti and Bocchini, 1995). It is possible to combine pyrolysis GC/MS with thermogravimetric analysis (TGA) to determine the temperatures at which mass loss occurs as this gives complementary and relevant information of cell wall composition (Ghetti et al., 1996; Carrier et al., 2011; Greenhalf et al., 2012). Sampling by GC/MS is generally made during the thermal transitions identified by TGA; these signify the decomposition of discrete classes of cell wall components (Bridgeman et al., 2007). Pyrolytic methods provide rich orthogonal data compared to the methods more generally employed in the analysis of biomass, but obtaining quantitative data is often challenging, and the approach suffers from several potential weaknesses: Firstly, sample amounts are small (~10 mg), and it is essential that they are representative. Secondly, the method is indirect in that it measures and identifies thermal degradation products rather than the cell wall components themselves, and relating these breakdown products to their parent compounds in the tissue sample is not trivial. Lignin thermal decomposition products are comprised of aromatic moieties with or without alkyl substitution, and many have been identified and associated with lignin (del Rio et al., 2001; Fahmi et al., 2007b). In contrast, structural carbohydrates degrade into furanones and pyranones by sequential dehydration processes, and these products are more difficult to ascribe to particular origins (Galletti and Bocchini, 1995).

#### Infrared and raman methods

Over recent years, many researchers have developed methods to analyse the cell wall based on near infrared reflectance spectrophotometry (NIRS), Fourier transform mid-infrared spectrophotometry (FTIR) and Raman spectroscopy. Spectral methods are generally simple in execution, non-destructive and are often rapid compared to other methods. Infrared (IR) and Raman spectroscopy provide information on molecular bonds present in the samples. For analysis by FTIR spectroscopy, which informs on fundamental molecular vibrations, samples are generally dried to eliminate water, which has high absorption in the IR. FTIR analysis generally requires a small amount of sample, which traditionally was finely ground biomass mixed with an IR-transparent salt, e.g., KBr, and pressed into transparent discs using 5–10 tonnes of pressure (Kačuráková et al., 2000; Xu et al., 2007a). FTIR spectroscopy has been used to study the structure of pectin and hemicellulose (Kačuráková et al., 2000; Sun and Tomkinson, 2002; Xu et al., 2006a, 2007a), cellulose (Liu et al., 2006), lignin (Sun et al., 2002a,b; Gosselink et al., 2004; Xu et al., 2007b) and pyrolysis char (Hu et al., 2008). These studies used small numbers of samples, and the time-consuming step of pressing salt disks presented no problems. More rapid methods of sample spectral analysis, such as attenuated total reflection (ATR) are more applicable for studies requiring larger numbers of samples. Furthermore, the flexibility of ATR is such that it allows the analysis of aqueous samples (Gosselink et al., 2004; Allison et al., 2009b). We recently used FTIR spectroscopy and partial least squares regression to predict the concentration of lignin and hydroxycinnamic acids (Allison et al., 2009b), nitrogen and alkali index (Allison et al., 2009a) in samples from a variety of grasses.

NIRS is an established method for analysis of biomass composition and many related parameters such as biomass recalcitrance and calorific value. Predictions are generally made by multivariate regression models, and the technique has been used to measure the concentration of lignin (Jung, 1997; Brinkmann et al., 2002; Takahashi et al., 2004; Robinson and Mansfield, 2009) and lignin monomer composition (Alves et al., 2006b), NDF and ADF (Jung, 1997; Pires and Prates, 1998; Petisco et al., 2006), digestibility (Nousiainen et al., 2004), nitrogen (Gislum et al., 2004), cell wall sugars (Sanderson et al., 1996), thermal decomposition (Lee et al., 2009), recalcitrance (Huang et al., 2012a) and heating value (Huang et al., 2007). Analysis is often performed on ~5 g of material held in a ring cell that is lowered into the spectrophotometer (Sanderson et al., 1996; Gislum et al., 2004).

In comparison, analysis of cell wall composition by Raman spectroscopy has until recently been uncommon due to high instrument cost, technical limitations, e.g., poor sensitivity and high sample fluorescence, as well as the incorrect assumption that Raman spectroscopy provides information that is more easily obtained by IR spectrometry. Newer instruments have overcome the technical limitations, and old prejudices have been replaced by the realization that Raman and IR spectral data are complementary (Stewart et al., 1997; Agarwal, 1999).

**Figure 1:**
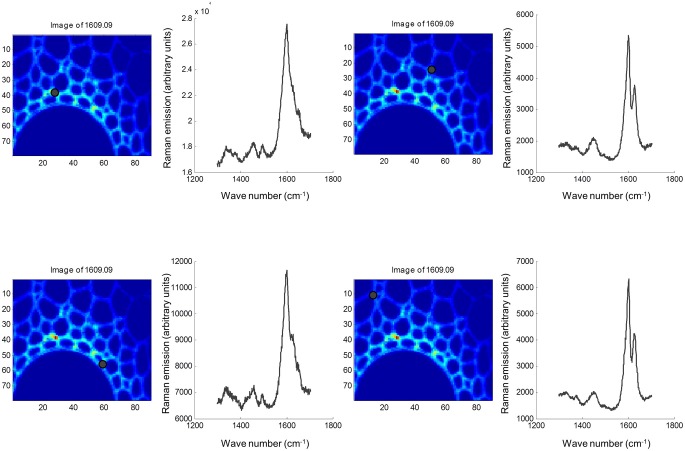
**Raman spectra at four locations in sections of mature maize stem internode.** Sections were fixed in glutaraldehyde, cut to a thickness of 2 μm and mounted on silicon slides (Bruker, Coventry, UK). Raman point spectra from 1300 to 1704 cm^−1^ were acquired using a Renishaw inVia Raman micro-spectrophotometer at ×50 magnification in 1-μm steps using a 514-nm green laser and an exposure time of 2 s/pixel. Spectral sampling at each of the four discrete locations are marked with black dots. Images were exported into Matlab (MathWorks, Cambridge, UK), and chemometric analysis was performed using the Eigenvector PLS and MIA toolboxes.

**Figure 2:**
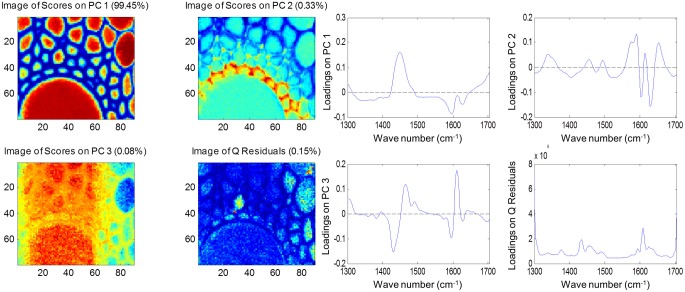
**Principal component analysis of Raman spectra maps of sections of mature maize stem internode.** Score images **(left)** and loading plots **(right)** are shown for the first three components together with the score image and loadings for the Q residuals (variance explained by each component and the residual is given in brackets). Spectral data were pre-treated using Savitsky-Golay smoothing (11-nm window, 1 polynomial), extended multiplicative scatter correction and mean centering.

**Figure 3:**
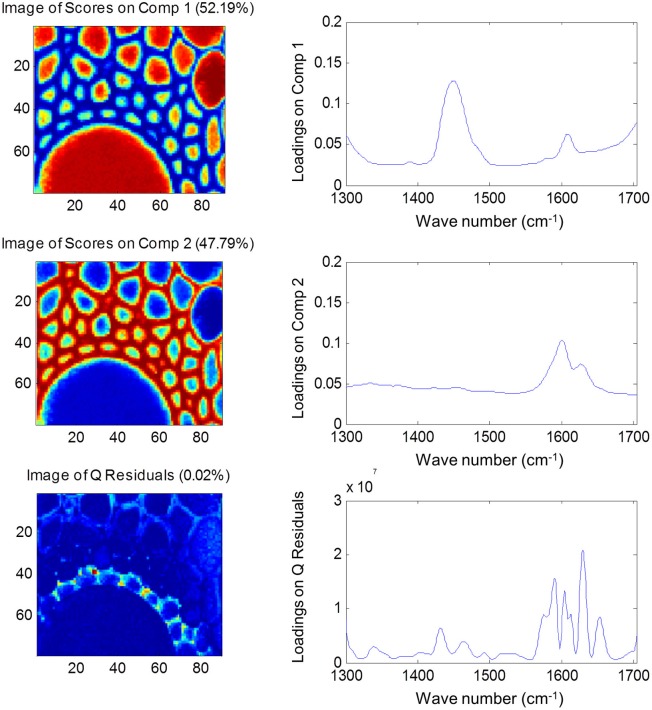
**Multiple component resolution analysis of Raman spectra maps of sections of mature maize stem internode.** Score images **(left)** and loading plots **(right)** are shown for the first two components, together with the score image and loadings for the Q residuals (variance explained by each component and the residual is given in brackets). Spectral data were pre-treated using Savitsky-Golay smoothing (11-nm window, 1 polynomial), extended multiplicative scatter correction and mean centering.

Both FTIR and Raman spectroscopy are highly suitable for microscopy. The former technique has been used for the identification and characterization of cell wall mutants (Chen et al., 1998; Mouille et al., 2003; McCann et al., 2007), and the time required for sample analysis has decreased substantially with the availability of array type detectors. However, the incompatibility of FTIR microscopy with the presence of water in the samples prevents the analysis of undried samples. Furthermore, the presence of fixatives used to dry samples prior to sectioning may create artifacts in the spectral data and spatial resolution is limited to 2–5 μm by the wavelength of mid IR light. Raman microscopy often circumvents many of these problems. Spatial resolution is frequently higher than 1 μm (Schmidt et al., 2010), and can be improved still further using modified approaches such as surface enhanced Raman spectroscopy (Agarwal and Reiner, 2009; Knauer et al., 2010; Scott and Carron, 2012). Furthermore, Raman spectroscopy is tolerant of water and the analysis of unfixed, cryogenically preserved, or polyethylene glycol impregnated samples is possible (Gierlinger et al., 2012). The resolution and flexibility of Raman imaging has been demonstrated recently in studies of the ultra-structure and composition of the cell wall in tree species and corn stover (Agarwal, 2006; Gierlinger and Schwanninger, 2006; Sun et al., 2011). Raman imaging has also been used to study delignification in samples of *Miscanthus* × *giganteus* treated with NaOH (Chu et al., 2010), and in our laboratory Raman imaging is being combined with multivariate image analysis tools to probe the architecture of the *Miscanthus* cell wall. We recently presented preliminary results from principal components analysis (PCA) and multivariate curve resolution (MCR) that we used to decompose hyperspectral Raman images of resin-embedded (LR white) sections of mature maize stem internode (Gordon Cell Wall Conference, Maine, USA, 2012). Figure 1 shows spectra taken at different positions and the differences between the spectra are most likely indicative of changes in lignin monomer ratio around the xylem vessel wall. PCA (Figure 2) indicated three orthogonal variance components, with the largest (PC1) being due to absence or presence of resin (colored red) and lignin (colored blue). The spectral differences attributed to differences in lignin monomer ratio accounted for a much lower portion of the total variance and are explained by PC2. PC3 and the Q residual explain even smaller portions of total variance and indicate variation in section thickness and noise, respectively. MCR offers an alternative approach to image analysis that is perhaps more subtle than PCA and offers components that, whilst not orthogonal, are of chemical significance as the algorithm is essentially a multivariate extension of Beer's Law. Analysis by MCR of the same Raman point maps (Figure 3) gave a clearer dissection of the spectral components in the image, with components 1 and 2 being resin and lignin, respectively. The variation in S/G ratio is likely shown in the residual. The presence of high loadings for this component around 1600 cm^−1^ supports this assumption. We are working to improve the resolution of specific cell wall components by investigating whether the prior training of the MCR model on spectra from high G and S polymers, or isolated cell wall carbohydrates, would improve spectral deconvolution and allow identification of discrete wall components in sections. The full potential of Raman imaging has yet to be discovered, but it is likely to be a key tool to the dissection of cell wall composition at the scale of cell wall architecture and allow the high precision functional characterization of cell wall genes and cell wall mutants.

## Genetic variation

**Figure 4:**
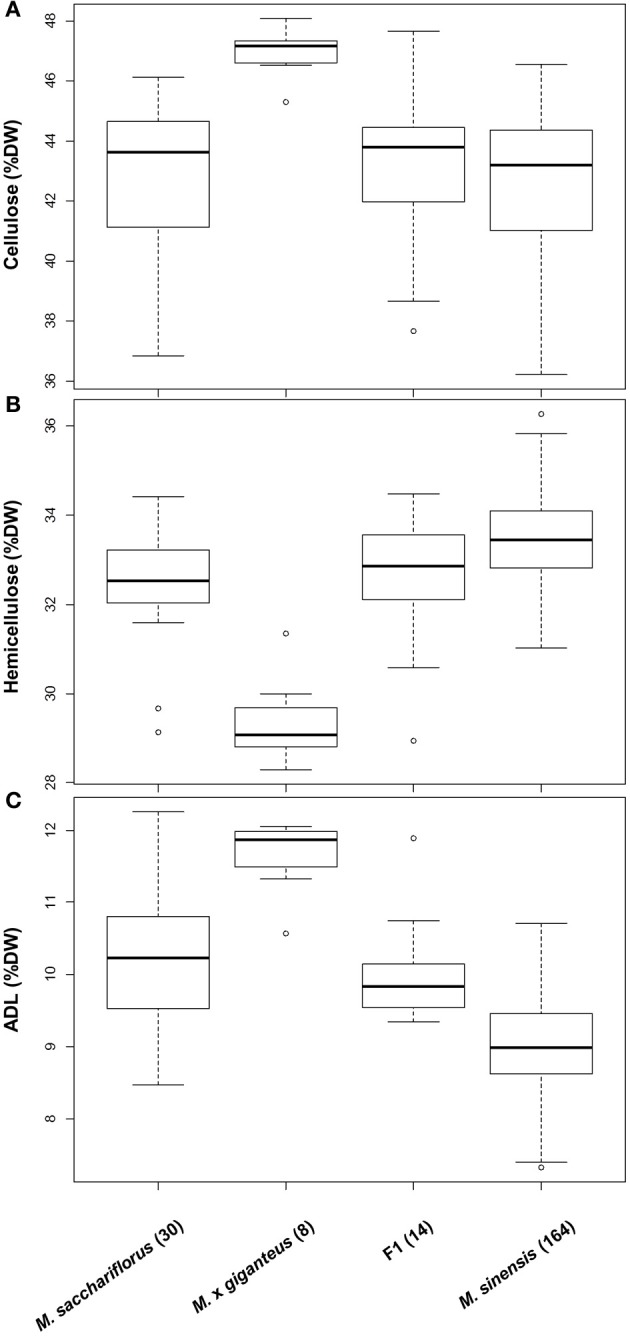
**Genetic variation for cellulose (A), hemicellulose (B) and lignin (C) contents in two *Miscanthus* species and their hybrids.** Data from a previous study (Allison et al., 2011) were re-analysed after classifying genotypes using 120 single nucleotide polymorphisms (Slavov et al., 2013). Distributions are based on least square means corrected for year and block effects (Allison et al., 2011). Boxes delineate inter-quartile ranges (IQR), whiskers extend to 1.5 × IQR, and thick lines correspond to medians. The number of genotypes in each group is shown in parentheses. ADL, acid detergent lignin; DW, dry weight; F1, hybrids with estimated *M. sinensis*: *M. sacchariflorus* admixture proportions between 1:2 and 2:1.

Consistent with findings for other phenotypic traits (Jensen et al., 2011; Robson et al., 2012; Slavov et al., 2013), extensive genetic variation for cell wall composition appears to be present (i) across *Miscanthus* species, (ii) among populations within species, and (iii) among genotypes within local populations (Hodgson et al., 2010b; Allison et al., 2011; Lygin et al., 2011; Slavov et al., 2013). At the inter-specific level, two general patterns were detected based on gravimetric measurements of NDF, ADF, and ADL of 244 genotypes grown in a field trial near Aberystwyth, UK (Allison et al., 2011). First, levels of hemicellulose and lignin differed subtly but significantly between *M. sinensis* and *M. sacchariflorus*, whereas distributions of cellulose content were statistically indistinguishable between the two species (Allison et al., 2011; Figure 4, *P* = 0.92). Second, *M.* × *giganteus* accessions differed dramatically from both *M. sinensis* and *M. sacchariflorus* for all three major cell wall components (i.e., higher cellulose and lignin and lower hemicellulose contents in *M.* × *giganteus*), with virtually no overlap of the distributions of genotypic means corrected for year and block effects (Figure 4, *P* < 0.00012). In an attempt to elucidate the causes of this striking contrast, we generated cell wall component distributions for 14 *M. sinensis* × *M. sacchariflorus* hybrids, whose admixture proportions were similar to those of the *M.* × *giganteus* accessions based on 120 single-nucleotide polymorphism (SNP) markers (Slavov et al., 2013). Interestingly, the distributions of all three major cell wall components in these hybrids were statistically indistinguishable from those in *M. sacchariflorus* (*P* > 0.7), with hybrids having higher lignin (*P* = 0.00001), lower hemicellulose (*P* = 0.006) and comparable cellulose contents (*P* = 0.75) relative to *M. sinensis* genotypes. Thus, the extreme cell wall compositions of *M.* × *giganteus* accessions are unlikely to be caused solely by the combination of *M. sinensis* and *M. sacchariflorus* genomes. Alternative explanations include highly unusual cell wall composition(s) of the progenitors of *M.* × *giganteus* and/or specific genome dosage effects (Yao et al., 2013) resulting from its presumed triploidy (Linde-Laursen, 1993; Swaminathan et al., 2010).

**Figure 5:**
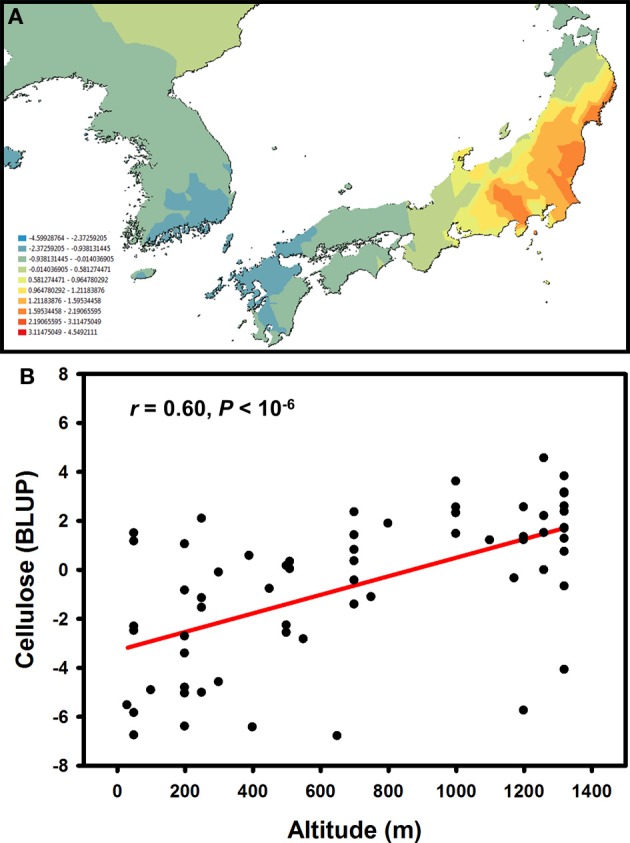
**Geographic pattern of genetic variation for cellulose content in a *Miscanthus sinensis* population (Allison et al., 2011; Slavov et al., 2013).** Spatially interpolated (i.e., kriged using Geostatistical Analyst in ArcMap 10, Esri Ltd., Aylesbury, UK) best linear unbiased predictors (BLUPs) of genotypic values for cellulose content in 2-year-old plants **(A)** and relationship between cellulose content BLUPs and accession source altitudes **(B)**.

Knowledge about intra-specific levels of genetic variation in *Miscanthus* is relatively limited (Głowacka, 2011), although on-going research and breeding programmes are rapidly accumulating detailed quantitative data. Taking advantage of a large germplasm collection available at the Institute of Biological, Environmental and Rural Sciences, we recently used a combination of SNP and microsatellite markers to objectively delineate population genetic structure in a set of *M. sinensis* genotypes (i.e., without *a priori* assumptions about the significance of geographic barriers or the spatial scale of genetic differentiation) and then juxtaposed geographic patterns of genetic variation for phenotypic traits with those for putatively neutral molecular markers (Slavov et al., 2013). These analyses indicated that the spatial distribution of genetic variation for major cell wall components was distinctly different from those for putatively neutral molecular markers and phenotypic traits related to phenology and biomass productivity. Molecular marker variation formed a clear longitudinal cline, with a genetic discontinuity defining “Continent” and “Japan” subpopulations (Slavov et al., 2013). In contrast, phenological and biomass traits, tended to correlate with source latitude and altitude, whereas multivariate measures of genetic variation for cell wall composition did not follow any simple geographic patterns. However, univariate analyses of genetic variation for cellulose content led to several intriguing findings. For example, cellulose content was much more strongly differentiated between the “Continent” and “Japan” subpopulations (*Q*_ST_ = 0.23−26) compared to neutral molecular markers (*F*_ST_ = 0.06). Furthermore, genetic variation for this trait followed clear spatial patterns both between and within the two subpopulations (Figure 5A). Interestingly, cellulose content increased with altitude (Figure 5B), whereas no concomitant reduction in hemicellulose or lignin was detected (Slavov et al., 2013). Taken together, these findings strongly suggest that genetic variation for cellulose content has been affected by spatially divergent selection. However, a formal tests of this hypothesis, as well as identification of the specific causes of selective diversification (e.g., key climatic variables driving cell wall composition along altitudinal gradients), would require carefully designed experiments using plant materials from multiple regions of the geographic range of *M. sinensis* and a diverse set of test environments.

Despite the striking species differences and significant inter-population differentiation, much of the genetic variation across a range of phenotypic traits, including cell wall composition, is found among genotypes within a population. Even for traits that have putatively been affected by divergent selection (e.g., cellulose, see above), within-population genetic variance components are roughly an order of magnitude greater than those among populations (Slavov et al., 2013). Because of the extensive genetic variation found in *Miscanthus* at multiple taxonomic and spatial scales, as well as the ability to capture this variation through carefully designed field trials and phenotyping protocols (i.e., broad sense heritabilities for cell wall composition traits ranged from 0.52 to 0.79 in *M. sinensis*, Slavov et al., 2013), a range of approaches to dissecting the genomic architecture of cell wall biosynthesis should be highly effective.

## From phenotype to genotype

Although the availability of functional genomics resources for *Miscanthus* is currently limited (see Section Molecular biology of cell wall biosynthesis), a variety of tools for dissecting the genomic architectures of phenotypic traits are rapidly developing. In this section, we will review the prospects of applying (i) quantitative trait locus (QTL) mapping in full-sib families resulting from controlled bi-parental crosses; (ii) genome-wide association studies (GWASs), in which high-density arrays of molecular markers are used to scan for genotype-phenotype associations in populations of putatively unrelated individuals; and (iii) admixture mapping (i.e., a set of analytical approaches that use the properties of populations comprised of individuals with mixed ancestries to identify phenotypic associations) to elucidate the molecular underpinnings of cell wall structure and recalcitrance in *Miscanthus*. However, various combinations of these approaches are also possible and could potentially be more powerful.

### QTL mapping

Early linkage mapping efforts in *M. sinensis* were based on Randomly Amplified Polymorphic DNA (RAPD) markers (Atienza et al., 2002) and were instrumental for the detection of QTLs for a number of phenological, agronomic, biomass productivity and composition traits, including components that affect combustion quality (Atienza et al., 2003a,b,c,d,e). However, because of the relatively small size of the mapping population used in these studies (*N* = 89), relatively little was learned about the genomic architectures of the traits of interest, and estimates of QTL effect sizes are likely to be very optimistic (Beavis, 1994; Xu, 2003), rendering the applicability of these results for marker-assisted selection (MAS) unclear. Recent advances in sequencing and genotyping technology (Davey et al., 2011), combined with the realization that larger mapping populations are needed to achieve adequate statistical power, are significantly enhancing the prospects of detecting more complete sets of QTLs in *Miscanthus*. For example, substantially denser and higher-quality linkage maps are currently available for both *M. sinensis* and *M. sacchariflorus* (Kim et al., 2012a; Ma et al., 2012; Swaminathan et al., 2012). All of these maps are anchored to the *Sorghum bicolor* genome (Paterson et al., 2009), which enables the interpretation of QTL mapping results and provides a means for comparative genomic studies. Furthermore, dozens of inter- and intra-specific mapping families, some of which as large as *N* = 1000, are currently developed and planted at multiple locations in Europe and the US. For example, preliminary analyses of the family used to produce the highest-density map that is currently available for *Miscanthus* (Ma et al., 2012) have resulted in the identification of tentative QTLs for a wide range of phenotypic traits, including major cell wall components, simple carbohydrate contents and various measures of recalcitrance (X.-F. Ma and T. Swaller, personal communication). In many cases, alignment of these QTLs to the *Sorghum* genome and cross-reference with QTL or GWAS data from other grasses results in the identification of manageable numbers of plausible candidate genes. In summary, linkage mapping is likely to be a major facilitator of biological discovery in *Miscanthus*, and the next generation of QTL mapping results will play a key role in depicting the genomic complexity of phenotypic traits. However, a major challenge for this approach is the need to verify the significance of QTLs outside of the family used for their detection.

### GWASs

**Figure 6:**
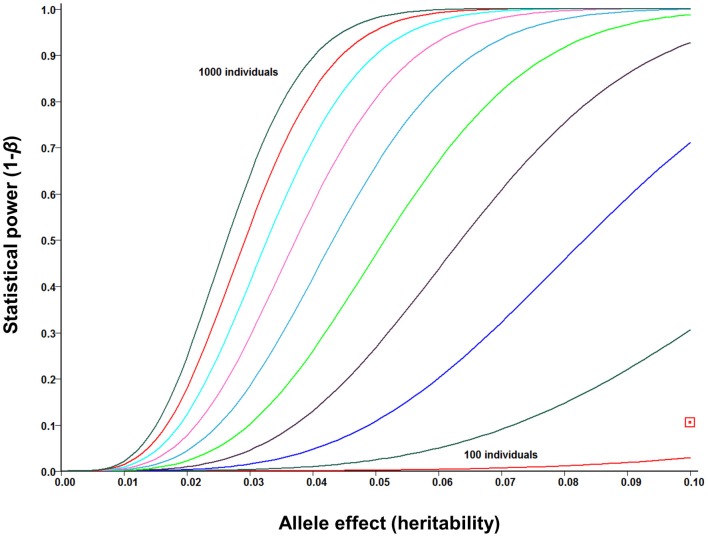
**Statistical power of genome-wide association studies (GWASs) under ideal circumstances (i.e., no confounding, causative polymorphisms genotyped directly) for mapping population sizes ranging from *N* = 100 to *N* = 1000 (in increments of *N* = 100) and allele effect sizes (i.e., proportion of phenotypic variance explained by each allele) up to 0.10.** Power curves were calculated using the GWAPower programme (Feng et al., 2011). Red square: power of pilot scale GWAS population in *Miscanthus sinensis* (*N* = 142).

The increasing affordability of SNP genotyping, and especially genotyping-by-sequencing technology (Elshire et al., 2011; Poland et al., 2012), is rapidly changing the status of GWASs from a “luxury good” reserved for model organisms to a standard genetic tool that can be used both for answering fundamental biological questions and for accelerated crop improvement (Hamblin et al., 2011; Morrell et al., 2012). In addition to *Arabidopsis* (Atwell et al., 2010; Filiault and Maloof, 2012) and major cereal crops (Buckler et al., 2009; Huang et al., 2010, 2012c), this approach was recently applied in *Sorghum* (Morris et al., 2013), the closest relative of *Miscanthus* for which genome sequence is available. Several lessons have been learned from early GWASs. First, while potentially very powerful, this approach tends to be susceptible to both genetic and environmental confounding (Atwell et al., 2010; Huang et al., 2012c; Vilhjalmsson and Nordborg, 2013). Proposed solutions to this problem range from various statistical approaches to account for population structure and relatedness (Balding, 2006; Yu et al., 2006; Price et al., 2010) to designing synthetic association mapping populations (Yu et al., 2008; Kover et al., 2009), which combine the strengths of GWASs (i.e., ability to screen a broad genetic base) and QTL mapping (i.e., robustness to confounding by population structure). Second, population sizes in the thousands are likely to be needed to provide a complete picture of the genomic architectures of phenotypic traits. This is because the majority of phenotypic associations explain relatively low proportions of the genetic variation (Li et al., 2012), whereas GWASs are inherently underpowered to detect small-effect associations (Figure 6) and/or associations with rare variants (Bansal et al., 2010; Cirulli and Goldstein, 2010; Gibson, 2012). Finally, consistent with findings from human GWASs (Hindorff et al., 2009; http://www.genome.gov/gwastudies/), the majority of trait-associated SNPs in maize were outside of coding regions, with 5-kb putative promoter regions upstream of genes being the most enriched category (Li et al., 2012). An important implication of this result is that RNA-seq approaches, while potentially very informative for a range of biological questions (Ozsolak and Milos, 2011), may not be the most appropriate genotyping tool for GWASs. Sequence capture, genotyping-by-sequencing and low-coverage whole-genome re-sequencing appear to be the most promising low-cost alternatives (Davey et al., 2011).

With these lessons in mind, we are designing GWAS populations for the three *Miscanthus* species that are expected to be most important for breeding programmes in Europe and the US (i.e., *M. sinensis*, *M. sacchariflorus* and *M. floridulus*). For example, we recently used a combination of microsatellite and SNP markers to delineate a proof-of-concept GWAS population in *M. sinensis* based on its relatively low levels of substructure and high levels of genetic variation for phenotypic traits related to phenology, biomass productivity and cell wall composition (Slavov et al., 2013). Subsequently, we collaborated with Floragenex, Inc. to genotype 142 individuals from this population using RAD-Seq technology (Baird et al., 2008) and the *PstI* restriction enzyme. Following stringent filtering based on alignment statistics and conformity of genotype frequencies to Hardy-Weinberg proportions, we identified 20,127 SNPs resulting from alignments to *Sorghum* and 30,755 resulting from alignments to *de novo Miscanthus* assemblies around *PstI* sites. We then used these data for pilot-scale GWASs and genome-wide phenotype prediction for traits related to phenology, biomass productivity and cell wall composition G. T. Slavov (personal communication). To control for the confounding effects of population structure and relatedness, we used the efficient mixed linear model (MLM) approach implemented in the EMMAX programme (Kang et al., 2010). More specifically, we tested the effect of each individual marker based on an MLM including the Identity-By-State (IBS) matrix and the primary eigenvector of population structure (Patterson et al., 2006), which were estimated based on the entire set of markers. This approach is widely used and regarded as the most robust and statistically powerful (Price et al., 2010; Vilhjalmsson and Nordborg, 2013), although the ever increasing volumes of data will likely require substantial improvements of its computational efficiency (Svishcheva et al., 2012).

As expected from the small population size, our pilot-scale GWAS is severely underpowered (Figure 6), and none of the SNP-trait associations for the cell wall composition data generated by Allison et al. (2011) reached genome-wide significance, after Bonferroni adjustment for multiple testing. However, several associations for each trait reached suggestive significance (*P* < 10^−4^), with many weaker associations aligning to plausible candidate genes in *Sorghum* (discussed in Section Functional genomics for enhanced cell wall deconstruction). Furthermore, preliminary results from cross-validations of genomic selection (i.e., phenotype predictions using all available markers, rather than identifying significant associations) in *M. sinensis* are encouraging, with estimated prediction accuracies (i.e., correlations of predicted genetic values with the unobservable “true” genetic values) of 0.69, 0.48, and 0.47, respectively, for cellulose, hemicellulose, and lignin contents G. T. Slavov (personal communication). Thus, the application of these approaches to larger populations and higher densities of markers looks very promising.

### Admixture mapping

Mapping by admixture linkage disequilibrium (MALD) was originally proposed as a way of performing genome scans in highly diverse populations using relatively small numbers of molecular markers (Chakraborty and Weiss, 1988; Smith and O'brien, 2005). However, recent statistical refinements and extensions of this approach, and particularly its application based on GWAS data, have made it a potentially very powerful tool for the dissection of complex phenotypic traits (Seldin et al., 2011). Although admixture mapping has been used to address fundamental questions in plant evolutionary biology (Buerkle and Lexer, 2008; Lexer et al., 2010; Stölting et al., 2013), as well as for trait mapping and improvement in breeding populations (Humphreys et al., 1997, 2005; Kosmala et al., 2007), its potential has been underutilized. The presumed occurrence of *M. sinensis* × *M. sacchariflorus* natural hybrid zones (Nishiwaki et al., 2011), and particularly the strong interest in generating inter-specific hybrids as part of breeding efforts, will likely make this approach more important in the future, although its successful application will hinge on the detailed characterization of genetic structure and linkage disequilibrium in reference populations of pure *Miscanthus* species.

## Molecular biology of cell wall biosynthesis

The plant cell wall is a complex heterogeneous matrix, and a considerable portion of the plant genome encodes for proteins involved in the biosynthesis, deposition, remodeling and regulation of the various cell wall components. The heterogeneity of cell wall matrices is increased by the different structural and functional properties required during plant development and between different tissue and/or cell types. This necessitates the carefully orchestrated expression of many different cell wall related genes. Despite the importance of cell wall properties for the usefulness of *Miscanthus* as a feedstock for bioenergy and bioproducts, there is relatively little data on cell wall biology, genetics and chemistry in this genus. Most studies seem to focus on physiological and agronomical traits, as well as environmental and socio-economic aspects of growing *Miscanthus* as an energy crop (Clifton-Brown et al., 2007; Wang et al., 2008; Jensen et al., 2011; Cadoux et al., 2012; Maughan et al., 2012; Robson et al., 2012). Analysis of *Miscanthus* biomass mostly focuses on its chemical and physical characteristics, when used either directly for combustion or for the various pretreatment and conversion routes to produce bioenergy and products (Yoshida et al., 2008; Hodgson et al., 2011; Brosse et al., 2012; Guo et al., 2012; Huang et al., 2012b; Robbins et al., 2012).

Chemical composition data is currently limited for *Miscanthus*, most reports providing a relatively broad compositional analysis of the main cell wall components (cellulose, hemicellulose and lignin) of senesced plant material (Allison et al., 2011; Kim et al., 2012b). Perhaps the most detailed cell wall analysis to date is described by Lygin et al. (2011) in which the cell wall biomass of fully senesced tillers from five *M. sinensis* genotypes as well as *M.* × *giganteus* was analysed. Abundance of lignin and ether-bound phenolics were reported as the main determinants of lignocellulosic saccharification (Lygin et al., 2011), although regression coefficient values were rather low, particularly when considering that cell wall composition data for one switchgrass and one giant reed (*Arundo donax*) genotype were included in the association analysis. Clearly, more studies focusing on the biological, chemical and physical properties of the *Miscanthus* cell wall, including the variation of these properties between different genotypes and developmental stages, are required to improve our understanding of the diverse structural features of the *Miscanthus* cell wall. Integration of this knowledge with (i) data on cell wall deconstruction and conversion and (ii) expression patterns of cell wall related genes will be essential for formulating robust strategies aimed at improving lignocellulosic biomass quality traits in *Miscanthus*. The establishment of a detailed gene-expression atlas, similar to what is available for maize (Sekhon et al., 2011) and switchgrass (Zhang et al., 2013), combined with an associated chemical composition atlas for the most widely used *Miscanthus* species (*M. sinensis*, *M. sacchariflorus* and *M. floridulus*) would provide a useful resource for the scientific community working on improving various traits in the *Miscanthus* biofuel crop. In addition, a *Miscanthus* gene-expression atlas, in conjunction with those available in related grasses, would enable gene co-expression network analyses. This approach could result in the identification of modules of highly correlated genes that are potentially involved in related biological processes underlying agronomically, physiologically and biochemically important traits, thereby allowing for targeted hypothesis-based testing of gene-function relationships.

### Translational genomics

Although the genetic and genomic resources for *Miscanthus* are projected to increase over the next few years, translational genomics based on information from model species represents a major route to accelerating the improvement of desirable traits in this undomesticated bioenergy crop. It is generally recognized that *Arabidopsis* is not the most appropriate model for the study of cell wall related traits in the grasses. Probably the strongest argument for this is that the different types and abundances of hemicelluloses and phenolics in secondary cell walls of grasses result in a three-dimensional polymer network with different cross-linking properties, and hence deconstruction requirements, when compared to those of dicots. *Miscanthus* belongs to the grass subfamily Panicoideae and falls within the tribe of the Andropogoneae, together with important crops such as maize (*Zea mays*), sorghum (*Sorghum bicolor*) and sugar cane (*Saccharum officinarum*). A second tribe belonging to the Panicoideae contains, amongst others, switchgrass (*Panicum virgatum*) and several millet species, including pearl millet (*Pennisetum glaucum*) and foxtail millet (*Setaria italica*). Among grasses with sequenced genomes, sorghum has the closest phylogenetic relationship to *Miscanthus* followed by that of maize (Ma et al., 2012; Swaminathan et al., 2012). This and the molecular and genetic tools available for sorghum and, in particular, maize make these two C_4_ crops good model systems for gene-discovery studies relating to cell wall and other relevant biomass traits for bioenergy grasses, including *Miscanthus* (Lawrence and Walbot, 2007; Carpita and McCann, 2008; Bosch et al., 2011; Calvino and Messing, 2012). However, validation of candidate gene function is not straight-forward and is relatively time-consuming in maize and sorghum, particularly when compared to functional analysis in the *Arabidopsis* model system.

Over the last few years, the grass *Brachypodium distachyon* has been increasingly employed as a model for bioenergy crops as it contains a number of attributes that make it a good system for functional genomic studies in the grasses (Draper et al., 2001; Brkljacic et al., 2011). *Brachypodium*, which uses the C_3_ photosynthetic pathway, belongs to the grass subfamily Pooideae, together with important agronomical crops such as wheat, oat, rye and temperate forage grasses. However, the more distant phylogenetic relationship between *Brachypodium* and energy grasses should not represent a barrier for its usefulness as a model for studying cell wall related traits. Transcript profiling of leaves from closely related C_3_ (*Cleome spinosa*) and C_4_ (*Cleome gynandra*) species showed that few cell wall related genes were differentially expressed (Brautigam et al., 2011). One exception was the higher expression of three transcripts encoding for glycosyl hydrolase family 17 1,3-β-glucosidases in the C_4_ species. These genes are possibly involved in governing plasmodesmatal conductivity by regulating the turnover of the β-1,3-glucan callose (Levy et al., 2007). Detailed analysis and comparison of cell wall characteristics between a number of C_3_ (*n* = 6) and C_4_ (*n* = 5) grasses showed no consistent patterns for differences in lignin content and composition, *p*-coumaric acid and ester-linked ferulic acid content, carbohydrate composition and sugar release (Hatfield et al., 2009). For most characteristics, variation within the C_3_ or C_4_ type grasses was higher than that between the two types. This indicates that C_3_ grasses can be used as a model for cell wall studies in C_4_ energy grasses. It also highlights the additional requirement for detailed biochemical and molecular analysis of cell wall characteristics in each individual bioenergy crop.

The adoption of *Brachypodium* as a model is nourished by the growing number of genetic resources and molecular toolkits available (Brkljacic et al., 2011; Mur et al., 2011), including a T-DNA mutant collection, albeit currently only covering a limited number (<10%) of the annotated *Brachypodium* genes (Bragg et al., 2012). The functional characterization of a cell wall related T-DNA mutant has not yet been reported. Screening of a chemically induced *Brachypodium distachyon* mutant collection identified mutations in the cinnamyl alcohol dehydrogenase 1 gene (BdCAD1) involved in lignin biosynthesis. Mutant plants showed reduced levels of lignin, altered lignin structure and, importantly, an over 40% improvement in saccharification efficiency, without compromising biomass yield (Bouvier D'yvoire et al., 2013). This shows that mutant collections provide a valuable resource for reverse genetic screens to identify cell wall related genes and associated function. Other resources to study cell wall related traits in *Brachypodium* include recombinant inbred line (RIL) populations (Cui et al., 2012) and germplasm collections comprising of genotypes collected from different geographies and ecological niches (Mur et al., 2011).

*Brachypodium*, like *Arabidopsis*, can be considered a model species as it has little agronomic value. However, as a result of on-going technological advances in plant genomics and phenomics, the distinction between model grasses and agronomically and economically important crops is slowly fading as more tools become available to study traits directly in relevant crop species. Maize and rice probably represent the best examples for this, but genomic resources for other crops such as wheat and barley are rapidly expanding (Brenchley et al., 2012; Consortium, 2012). Reference genome sequences have also recently become available for foxtail millet (Bennetzen et al., 2012; Zhang et al., 2012). In addition to having potential as a C_4_ biofuel crop in its own right, foxtail millet can serve as a model system for other biofuel grasses. An important step for future research is to validate the transferability of molecular biological findings related to cell wall biology in model grasses to the more genetically recalcitrant, and therefore challenging, bioenergy crops such as *Miscanthus*.

### Functional genomics for enhanced cell wall deconstruction

The fact that *Miscanthus* is a new crop lacking the history of extensive breeding and research, e.g., as in wheat and maize, combined with its large genome size (~2.5 Gbp; Swaminathan et al., 2010) and complexity of genome structure, provides significant challenges for trait improvement. The biggest asset for the domestication of *Miscanthus* as a sustainable energy crop is the genetic and phenotypic diversity present within and among *Miscanthus* species (see Section Genetic variation).

A pilot scale GWAS in 142 *M. sinensis* genotypes (see Section GWASs) identified hundreds of SNPs that were at least weakly associated (*P* < 0.05) with gravimetrically measured cellulose, hemicellulose and lignin contents (Allison et al., 2011). More than 44% of the SNPs that were tentatively associated with cellulose content (*P* < 0.05) were identical between two years of cell wall composition measurement. This percentage was 40% for lignin content, but significantly lower (19%) for hemicellulose, possibly reflecting the genetic and structural complexity of hemicelluloses. As discussed in Section GWASs, the statistical power of this pilot scale GWAS experiment is very limited (Figure 6), and results should therefore be approached with great caution. Nevertheless, some promising and interesting findings can be distilled from this study. As an example, tentative associations with cellulose content included a SNP located in a putative *Miscanthus* ortholog of MYB46 in *Arabidopsis* (AtMYB46), maize (ZmMYB46), and rice (OsMYB46). All of these have been shown to act as master regulators for secondary cell wall formation (Ko et al., 2011) and references therein. Interestingly, a recent study has shown that AtMYB46 directly regulates all three secondary cell wall associated cellulose synthase genes in *Arabidopsis* (Kim et al., 2013). As expected, over-expression of AtMYB46 results in a significant increase of crystalline cellulose content in *Arabidopsis*, indicating that this transcription factor is a good target for altering cell wall content in energy crops. Another interesting category of cellulose related SNPs were those found in genes for which the putative *Arabidopsis* orthologs are involved in vesicle mediated transport and organization of the microtubules. Cellulose microfibrils are synthesized by plasma membrane-localized cellulose synthase (CESA) complexes that move along cortical microtubules. Exocytosis of CESA proteins, most likely already complexed, to the plasma membrane takes place through Golgi derived vesicles (Crowell et al., 2009; Bringmann et al., 2012). The microtubule cytoskeleton influences the pattern and rate of cellulose biosynthesis by regulating the delivery of the synthesizing enzymes to the plasma membrane (Bringmann et al., 2012). Associated SNPs identified include the Golgi localized phosphoinositide phosphatase AtSac1 which is, amongst others, required for normal secondary cell wall synthesis and is therefore likely involved in the intracellular trafficking required during cell wall deposition (Zhong et al., 2005; Wightman and Turner, 2010). The AtSac1 mutant, *fragile fiber 7* (*Fra7*), shows a dramatic decrease in the wall thickness of fiber cells and vessel elements, which seems partly caused by a reduction of crystalline cellulose (Zhong et al., 2005). Another fragile fiber related gene, FRA1, with an associated SNP for cellulose content in *M. sinensis*, encodes for a kinesis-like motor protein. FRA1 is involved in the patterning of cellulose microfibrils as the mutant seems to specifically alter the orientation of cellulose microfibrils associated with a reduction in the mechanical strength of fibers (Zhong et al., 2002). More recent analysis of FRA1 has confirmed that it is a functional motor protein with the potential to drive long-distance transport of cell wall related cargo along cortical microtubules (Zhu and Dixit, 2011). Another SNP is associated with a gene encoding for a microtubule organization protein. Mutation in the *Arabidopsis* homolog, MOR1, resulting in microtubule fragmentation, leads to increased cellulose crystallinity (Fujita et al., 2011). Other cellulose associated SNPs for transport related proteins identified includes a putative homolog for AtSec20, which based on module-based predictions for functionally unknown genes in *Arabidopsis* was classified as being involved in Golgi vesicle transport and cellulose biosynthesis (Heyndrickx and Vandepoele, 2012). Thus, genes encoding for proteins involved in the transport/deposition of cell wall components might represent an interesting, and often overlooked, target for further biomass improvement through breeding and/or genetic engineering approaches.

### Genetic engineering for enhanced cell wall deconstruction

*Miscanthus* is an undomesticated crop and the genetic and phenotypic diversity available represents a good platform for using next generation sequencing (NGS) technologies and high-throughput trait assessments to accelerate breeding cycles. However, the urgent need to develop sustainable energy sources and mitigate climate change, combined with the complexity of cell wall related traits, requires additional approaches to rapidly deliver sustainable energy crops that are economically viable. Genetic manipulation, also referred to as genetic engineering, has the potential to significantly speed up the process of developing and improving *Miscanthus* varieties.

In contrast to other leading bioenergy crops, such as switchgrass, no comprehensive genetic engineering approaches have been reported thus far for *Miscanthus*. *Agrobacterium*-mediated genetic transformation of switchgrass is well-developed, and both the feasibility and benefits of genetic engineering approaches have been demonstrated by attempts to overcome cell wall recalcitrance through genetic interventions in the monolignol biosynthetic pathway. Down-regulation of the cinnamyl alcohol dehydrogenase (CAD) gene (Fu et al., 2011a; Saathoff et al., 2011) and caffeic acid O-methyltransferase (COMT) (Fu et al., 2011b) has shown that a reduction of lignin resulted in a concomitant reduction of cell wall recalcitrance as sugar release from the lignocellulosic biomass was improved. In the latter study, it was also demonstrated that the reduction of cell wall recalcitrance allowed using milder pretreatment and lower cellulase dosage to obtain equivalent ethanol yields to control biomass. Importantly, transgenic switchgrass plants in these studies looked phenotypically normal, except for the specifically targeted traits. This clearly demonstrates the potential for a significant cost reduction in the conversion of biomass to ethanol by implementing genetic engineering approaches to overcome cell wall recalcitrance.

Transcription factors represent attractive targets for the manipulation of complex metabolic pathways in plants, including the pathways leading to the synthesis of the different cell wall components, to improve lignocellulosic biomass traits. Several transcription factors are known to regulate secondary cell wall synthesis and therefore should provide useful tools for altering lignocellulosic biomass characteristics (Wang and Dixon, 2012). The overexpression of a switchgrass MYB transcription factor, PvMYB4, predicted to be an ortholog of AtMYB4 and ZmMYB31 (both transcriptional repressors of monolignol biosynthetic genes), resulted in reduced lignin content and ester-linked *p*-coumaric acid: ferulic acid ratio, and a 3-fold increase in sugar release efficiency from cell wall biomass (Shen et al., 2012b). However, plant development and architecture were affected in these transgenic plants. While representing good targets for changing the flux through cell wall synthesis related pathways, the complexity and potential pleiotropy of transcriptional regulation (Broun, 2004) should be carefully considered. As an example, overexpression of the *Arabidopsis* NAC transcription factor AtLOV1 in switchgrass delayed, as expected, flowering time but also led to the formation of erect leaves and increased lignin content, as well as altered monolignol composition with an increased guaiacyl:syringyl ratio (Xu et al., 2012).

The potential for more ingenious genetic engineering approaches to manipulate cell wall composition and recalcitrance has been illustrated by a recent study in which a synthetic biology approach was adopted in *Arabidopsis* to alter the deposition of cell wall polymers in the secondary cell wall (Yang et al., 2012). The spatial deposition patterns of lignin and polysaccharides were reprogrammed by altering promoter-coding sequence associations for a number of key, well characterized genes, which led to a reduction of lignin content and enhanced polysaccharide deposition in fiber cells (Yang et al., 2012). This rewiring of secondary cell wall deposition more than doubled the sugar yields after enzymatic hydrolysis, without obvious growth penalties.

Another strategy by which genetic engineering can be used to improve cell wall deconstruction is through the *in planta* expression of thermo-stable cell wall degrading enzymes from microbial origin. Being inactive during normal plant growth conditions, temperature induced expression of such enzymes promotes hydrolysis of cell wall polymers after harvest, thereby rendering the lignocellulosic biomass more amenable for further deconstruction and conversion to biofuels (Jung et al., 2012). An elegant example is provided by a recent study in which a thermo-regulated xylanase was engineered and expressed in maize (Shen et al., 2012a). The introduction of a self-splicing bacterial intein disrupted the xylanase activity at normal growth temperatures, but a temperature-induced (>59°C) splicing reaction restored xylanase activity. Consequently, mild heat pretreatment of transgenic corn stover, which induced *in planta* xylanase activity, led to a significant increase in sugar release, with the potential to reduce the production costs associated with biomass pretreatment and enzymatic hydrolysis.

Plant genetic engineering is not only important to increasing our understanding of the structure, function and synthesis of plant cell walls but also provides a route that can contribute to reducing the costs of lignocellulosic biomass conversion. Although genetic transformation of *Miscanthus* using particle bombardment (Wang et al., 2011) and *Agrobacterium*-mediated transformation (Engler and Jakob, 2013) has been reported there is, to our knowledge, no report on the functional analysis of transgenes expressed in *Miscanthus*. An efficient *Agrobacterium*-mediated transformation protocol, preferably for high yielding *Miscanthus* genotypes, needs to be developed to enable the effective improvement of key traits through genetic engineering approaches.

## Prospects

In addition to securing increased and stable biomass yields under a wide range of climatic and edaphic conditions, a crucial aim for energy crop scientists and breeders will be to enhance knowledge about the structure of cell walls and the key characteristics affecting the efficiency of the conversion of lignocellulosic biomass into fuels and products. The tremendous amounts of genetic variation among and within *Miscanthus* species, as well as the extensive heterosis observed in both natural and synthetic hybrids, reinforce the promise of this energy crop. Population genomics approaches appear to be particularly well-suited for harnessing this variation and informing both fundamental biology and breeding programmes. However, because the genomic architectures of most traits of interest appear to be exceedingly complex, bridging the statistical and biological gap between phenotype and genotype will likely require experimental population sizes in the hundreds and thousands. As with most other traits, phenotyping is likely to be the rate-limiting factor in this process. This is because none of the currently existing cell wall phenotyping approaches is sufficiently robust and scalable, although this field of research is extremely dynamic, and a variety of promising ideas are being developed and evaluated. In the short term, most of the progress in our understanding of the molecular underpinnings of cell wall quality traits in *Miscanthus* will be driven by research in model grasses. However, technological progress in the accuracy and throughput of cell wall chemotyping, integrated with the rapidly expanding genetics and genomics resources for *Miscanthus*, should provide exciting opportunities to discover and functionally test gene-trait associations for cell wall quality in this bioenergy crop.

### Conflict of interest statement

The authors declare that the research was conducted in the absence of any commercial or financial relationships that could be construed as a potential conflict of interest.
